# Vulnerability Factors Associated with Lifetime Posttraumatic Stress Disorder among Veterans 40 Years after War

**DOI:** 10.3390/healthcare8040359

**Published:** 2020-09-24

**Authors:** Ivone Castro-Vale, Milton Severo, Davide Carvalho, Rui Mota-Cardoso

**Affiliations:** 1 Medical Psychology Unit, Department of Clinical Neurosciences and Mental Health, Faculty of Medicine, University of Porto, 4200-319 Porto, Portugal; 2 Department of Clinical Epidemiology, Predictive Medicine and Public Health, and Department of Medical Education and Simulation, Faculty of Medicine, University of Porto, 4200-319 Porto, Portugal; milton@med.up.pt; 3 Department of Endocrinology, Diabetes and Metabolism, São João Hospital University Centre, Faculty of Medicine, University of Porto, 4200-319 Porto, Portugal; davideccarvalho@gmail.com; 4 i3S-Institute for Research and Innovation in Health, University of Porto, 4200-135 Porto, Portugal; rmc@med.up.pt

**Keywords:** posttraumatic stress disorder, war veterans, trauma and stressor related disorders, adverse childhood experiences, attachment

## Abstract

Vulnerability factors for posttraumatic stress disorder (PTSD) development are still controversial. Our aim was to study the vulnerability factors for the development of war-related PTSD over a period of 40 years after exposure. A cross-sectional, observational study was carried out on 61 male traumatized war veterans, taking into consideration adverse childhood experiences (ACE), attachment orientations, number of non-war-related traumatic events, and war experiences. Lifetime PTSD was assessed by using the Clinician-Administered PTSD Scale. Insecure attachment styles were significantly associated with lifetime PTSD and even after adjustment for war exposure this was still significant. Non-war-related traumatic events were not associated with lifetime PTSD, whereas ACE were associated with lifetime PTSD. War-related experiences were also associated with lifetime PTSD, except for injury or disease. The results for our sample show that, 40 years after war, the intensity of war-related experiences and ACE were significantly and independently associated with the development of lifetime PTSD. Insecure attachment was significantly associated with lifetime PTSD, which, in turn, are both positively associated with war exposure. These findings may have implications for patient care, as they constitute a strong argument that attachment-focused therapies could well be necessary 40 years after trauma.

## 1. Introduction

Posttraumatic stress disorder (PTSD) diagnostic criteria have recently been reviewed in the fifth edition of the Diagnostic and Statistical Manual of Mental Disorders (DSM-5) [1]. PTSD diagnosis requires the experience of a traumatic event (TE) to bring on its development. The TE definition has also changed from DSM-IV to DSM-5 criteria, with the latter not requiring the person to feel intense fear, helplessness, or horror—as DSM-IV does. It has been estimated that 9.2% of those exposed to a TE will have PTSD [2]. The reason why only a minority of exposed people come to develop PTSD is the focus of much research. Vulnerability factors have been described and can be grouped into three clusters. Pre-traumatic vulnerability factors have been found, such as: arousal, negative affect, hostility, anger, lower cognitive abilities, psychopathology, prior trauma, poor family functioning, poverty, and a family history of psychopathology. Among perceived peritraumatic vulnerability factors, life stress, emotional responses, and dissociation have all been found to predict PTSD development. Finally, it was found that lack of social support was the main posttrauma vulnerability factor for predicting PTSD development [3,4]. It has been found that peritraumatic factors are more predictive of posttraumatic growth, whilst pretrauma and personality-related variables are only predictive of PTSD [5]. Out of the personality variables, avoidant attachment significantly contributed to variance in PTSD risk.

Attachment is a construct which is related to the pattern of relationships established by a person with significant others. Attachment starts with the first relationships established between children and their caretakers, and is shaped by other relationships and events throughout the life cycle [6]. Attachment security influences the way a person copes with adversities and stress through positive mental representations of self and others [7]. On the other hand, insecure attachment orientations (anxiety and/or avoidance) predispose a person to mental disorders, due to the absence of a stable mental organization [7,8]. Attachment orientation predicts how adults react to stress and TEs [9,10]. Some studies suggest that attachment patterns moderate the association between TEs and PTSD [11,12]. Several attachment-related TEs are characterized by high conditional risk for PTSD development, or cause high PTSD burden to society, such as the sudden unexpected death of a loved one, war, sexual violence, and witnessing atrocities [2,13,14,15]. Furthermore, trauma severity has also been found to moderate the association between attachment and PTSD [16]. O’Connor and Elklit [17] found a negative correlation between secure attachment and PTSD symptoms when studying a non-clinical population, which suggested that secure attachment protects against the development of PTSD. This was also found in the case of combat-related PTSD [18,19]. On the other hand, PTSD was shown to influence attachment insecurity [20,21]. Furthermore, attachment has been shown to be negatively influenced by maltreatment as a child [22]. Attachment orientations can be studied as pretraumatic vulnerability factors for PTSD development, but also as peritraumatic, and even as a consequence of the disorder itself.

Among the pretraumatic vulnerability factors, adverse childhood experiences (ACE) have been related to the development of PTSD, with an increase of risk after exposure to the index TE [23]. Childhood adversities can challenge secure attachment organizations and have enduring consequences on attachment orientations and psychopathology [24,25]. ACE have also been associated with combat-related PTSD, particularly in the case of physical neglect and multiple types of adversities [26].

Different TEs are related to different incidence rates of PTSD, the highest rates being related to interpersonal violence [2]. War-related TEs are well-known risk factors for the development of PTSD, as well as traumatic load [27,28]. Specific combat experiences are associated with different risks to develop PTSD [29,30]. Some of these war experiences, such as atrocities and killing, may constitute severe transgressions of combatants’ deepest moral standards, and cause what Litz et al. [31] defined as ‘moral injury’—the long-term negative consequences at psychological, behavioral, religious, emotional, biological, and social levels. The study of war-related PTSD demands that war experiences are well characterized. However, the results regarding the influence of attachment orientations in the relationship between TEs and PTSD development are conflicting [9,11].

Portugal was involved in a war conflict from 1961 to 1974 with Angola, Mozambique, and Portuguese Guinea (currently Guinea-Bissau), which were former colonies fighting for their independence. Most of the soldiers were deployed non-voluntarily for a period of 24 months of guerrilla war.

Portuguese veterans of the colonial wars have a high prevalence (39%) of probable PTSD [32]. In addition, the time lapse of 40 years after the end of these wars provides a long after-trauma period for PTSD to develop, including delayed onset—a subtype of PTSD which applies to those cases when symptoms only begin six months or more after exposure to the TE [33]. Nevertheless, only a few studies have researched this population. Furthermore, the number of older war veterans is rising and, as the majority retain their diagnosis of PTSD following evidence-based interventions, and one third drop out of treatment, it is therefore important to characterize this population further [34]. The aim of this study was to investigate some of the vulnerability factors for lifetime PTSD development, over a period of 40 years after war-related TE, in a sample of Portuguese war veterans, especially focusing on ACE, attachment orientations, war experiences, and the experience of non-war-related TEs. In addition, we studied whether the association between war exposure and lifetime PTSD was confounded by or interacted with attachment orientations.

## 2. Materials and Methods

### 2.1. General Procedure

This cross-sectional, observational research is part of a larger study on the neurobiological inheritance of PTSD, which was approved by the Ethics Committee of our University (Comissão de Ética para a Saúde do Centro Hospitalar de São João/Faculdade de Medicina da Universidade do Porto, approval number: CES-138/08). Having received a complete written and verbal description of the study, all the participants gave their written informed consent. Interaction with the participants was carried out in a university setting and was solely performed by the same researcher and was carried out individually during one appointment. No financial compensation was paid for participating in this study, although payment for transport to the university was provided.

### 2.2. Participants

We used two ways of selecting participants (for a detailed description see Castro-Vale et al. [35]): 75.4% were from an outpatient clinic of the Portuguese Disabled Veterans Association (ADFA), and 24.6% were from three lists of war veterans’ companies from war time. Sixty-one male, Caucasian veterans (mean age of 65.25 (range = 60–74, SD = 3.37 years)) from the Portuguese colonial wars agreed to participate.

Participants with and without lifetime PTSD were included if they fulfilled the war-related DSM-IV [36] criterion A for PTSD, and also if they had children (as this is part of a larger study on the neurobiological intergenerational transmission of PTSD). The general exclusion criteria for participants were the following: the presence of neurologic, infectious, or any active medical illness, and any DSM-IV psychotic, bipolar, or neurocognitive disorders. Participants with lifetime PTSD were also excluded if they had current substance-related disorders. The specific exclusion criteria for the war veterans’ group without PTSD were the following: if they had ever had PTSD and if they also had any current psychiatric disorder.

### 2.3. Measures

The Graffar Index was used to measure the socioeconomic status (SES) [37,38]. This Index classifies subjects into five classes, with 1 being the highest, and 5 the lowest SES class. The clinical history and other sociodemographic data were also collected.

The Clinician-Administered PTSD Scale (CAPS) [39] was used to characterize the participants in relation to PTSD diagnosis, and also to research the lifetime number and the type of TEs experienced. Lifetime PTSD was considered if participants had DSM-IV criteria, in accordance with Blake et al.’s [40] rule (frequency ≥1 and intensity ≥2) and a total CAPS score of 50 or more. TEs were assessed with the CAPS Life Events Checklist and were subsequently checked for DSM-IV A2 criterion, following the CAPS procedure. TEs occurring before and after the war were counted separately from each other, and also from war-related TEs. In our sample, the Cronbach’s alpha (reliability) of the CAPS was superior to 0.90.

To determine participants’ eligibility requirements for the study, current and past psychiatric disorders where investigated, using the Structured Clinical Interview for DSM-IV axis I (SCID-I) [41] except for the PTSD module.

The Childhood Trauma Questionnaire-Short Form (CTQ-SF) [42,43] is a retrospective, self-reported questionnaire which contains 28 questions about specific maltreatment experiences during childhood and adolescence. The items are classified into a 5-point ordinal scale, according to the frequency of exposure to that specific experience. The Total CTQ-SF score provides a general ACE score, and not just TEs. It also provides scores for five different types of maltreatment, namely: emotional abuse; physical abuse; sexual abuse; emotional neglect, and; physical neglect.

Attachment style was studied with the Revised Adult Attachment Scale (RAAS) [44,45]. This scale consists of 18 items, which were scored on a 5-point Likert scale. The scale contains three dimensions: anxiety, close, and depend. The close and depend dimensions are positively correlated and can be gathered as close–depend. Close and depend dimensions were averaged and were then reverse scored to yield the attachment-related avoidance dimension. According to the scores obtained for each dimension, Bartholomew’s [46] attachment styles classification was adopted, namely: secure, dismissing, preoccupied, and fearful. These attachment styles are described as follows (adapted from [44]): secure—those participants who scored an average score below the midpoint (3.0) on avoidance, and below or equal to the midpoint on anxiety; dismissing—those whose scoring was above or equal to the midpoint on avoidance, and below or equal to the midpoint on anxiety; preoccupied—those scoring below the midpoint on avoidance, and above the midpoint on anxiety; fearful—those scoring above or equal to the midpoint on avoidance, and above the midpoint on anxiety. Those participants with dismissing, preoccupied, and fearful styles were also grouped in the insecure style [47].

In order to characterize and quantify the different war-related experiences of the veterans, we constructed the War Exposure Questionnaire (WEQ; see Supplementary Materials)—which was adapted to the specificities of the guerrilla war where each veteran fought. This questionnaire was adapted from the “Severity of Exposure Index”, which was used for the same purpose [32]. The WEQ has 38 items, which inquire about eight different subdomains of war-related experiences (war-related experiences, physical conditions, injury or disease, witnessing casualties amongst comrades, witnessing casualties amongst the enemy, witnessing casualties amongst civilians, actions on the enemy, and action against civilians). Each sub-domain is the result of the sum of positively-answered questions (e.g., have you been tortured?) which are related to that subject. The total sum provides a total WEQ score (ranging from 0 to 38), which represents war exposure, and which was used as a surrogate for war severity. In our sample, the Cronbach’s alpha (reliability) of the total WEQ score was 0.81.

### 2.4. Statistical Methods

The chi-square test, or the Fisher exact test was used to test the association between qualitative variables. The two independent sample *t*-test was used to compare the quantitative variables.

Odds ratio and the respective 95% CI was used to estimate the magnitude of the association between lifetime PTSD and several vulnerability factors. Simple and multinomial unconditional logistic regression was used to estimate the crude and adjusted odds ratio. The interaction between independent variables with lifetime PTSD was studied using logistic regression models.

## 3. Results

Participant’s characteristics are depicted in Table 1. Groups with, and without lifetime PTSD are identical with regards the subjects’ age, marital status, prevalence of disability, Graffar classification, and the deployment site where they were at war. All the veterans self-reported good physical and mental health before going to war.

**Figure 1:**
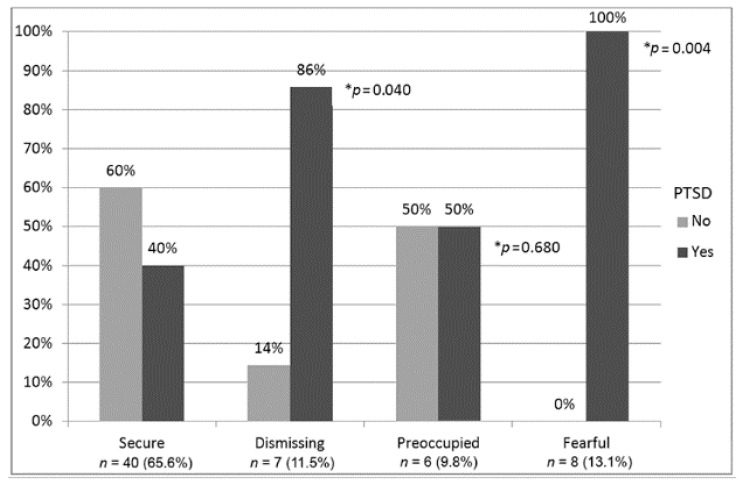
Prevalence of lifetime posttraumatic stress disorder (PTSD) by styles of attachment (*p* = 0.005). * Considering as reference, the secure style.

Attachment was significantly associated with lifetime PTSD (Table 2). High scores in anxiety and avoidance attachment were significantly associated with lifetime PTSD (*p* = 0.002 and 0.001, respectively). The association between anxiety and lifetime PTSD ceased to exist when adjusting for avoidance and total war exposure (total WEQ score). On the other hand, the association between avoidance and lifetime PTSD was attenuated, but still significant when adjusting for anxiety and total war exposure (*OR* = 7.21; 95% CI 1.02, 50.94; Table 2). When dimensions were converted to attachment styles, the prevalence of lifetime PTSD was significantly different between groups (*p* = 0.005; Figure 1). The groups with the dismissing (*p* = 0.040) and fearful (*p* = 0.004) styles of attachment have a higher prevalence of lifetime PTSD than the secure style group. A significant association was found between insecure attachment styles and lifetime PTSD (*OR* = 6.37; 95% CI 1.81, 22.46; Table 2). When adjusting for total war exposure, the association between insecure attachment styles and lifetime PTSD was attenuated, but was still significant (*OR* = 4.04; 95% CI 1.00, 16.34).

Having experienced non-war-related TEs (either before or after the war) was not associated with lifetime PTSD (Table 2). ACE, as assessed by the CTQ-SF, showed that total CTQ-SF experiences (*p* = 0.037), and particularly, emotional abuse (*p* = 0.024) and physical neglect (*p* = 0.004) were significantly associated with lifetime PTSD development (Table 3). Total CTQ-SF experiences remained significantly associated with lifetime PTSD after adjustment for total war-related experiences (*p* = 0.039). Furthermore, total CTQ-SF experiences did not interact with total war experiences to predict lifetime PTSD (data not shown).

With regards to war-related experiences, these were all significantly associated with lifetime PTSD, except for the case of those related to injury or disease (Table 3). Adjusting total war-related experiences to childhood adversities did not change the OR (Table 3), nor did adjusting for attachment dimensions (Table 2). Furthermore, total war experiences did not interact with attachment dimensions to predict lifetime PTSD (data not shown). In relation to action against civilians, 25 (49%) of the subjects who did not report this experience developed lifetime PTSD, whilst the nine (100%) who did report this experience developed lifetime PTSD (*p* = 0.013).

## 4. Discussion

We found that attachment orientations were associated with lifetime PTSD, particularly the insecure attachment styles. When adjusted for war exposure, this association decreased, but was still present. This means that war exposure is a confounder of the association between attachment and PTSD, or in other words, war exposure is associated with attachment and also with lifetime PTSD.

According to the attachment theory, insecure attachment is a risk factor for the development and increase in PTSD symptoms. However, Solomon et al. [21] found that PTSD symptoms predict attachment patterns better than attachment predicts PTSD symptoms. On the other hand, a longitudinal study concluded that PTSD symptoms both influenced, and were influenced by, attachment patterns, and that attachment insecurity contributes to maintaining PTSD symptoms over time [20]. Recent cross-sectional studies have found associations between attachment styles and war-related PTSD, however the design of these studies does not permit one to draw conclusions on causality e.g., [18,[19]. As our study does not also allow one to make conclusions about causality, longitudinal studies should be pursued in order to clarify these relationships further.

Considering Bartholomew’s [46] styles, in our study, the group of participants with the secure attachment style had a lower prevalence of lifetime PTSD than those with dismissing and fearful styles. These findings are probably related with the high association that we found between the avoidance dimension and PTSD. Clark and Owens [18] also found that the highest association with PTSD symptom severity was for avoidance attachment. As they argue, attachment avoidance may have some overlap with the avoidant and numbing PTSD symptoms, and it has been suggested that these increase across time [48]. A recent meta-analysis of the relationship between adult attachment style and post-traumatic stress symptoms found a modest overall population effect size for avoidant attachment [49]. In our study, all participants with the fearful style had lifetime PTSD. Other studies have found fearful style to be associated with the highest scores for PTSD symptoms (e.g., [19,49]). Studies of non-clinical and non-war-related PTSD samples also found associations between attachment and PTSD symptoms [50]—particularly associations with the dismissing and fearful styles [17,51]. Furthermore, a recent meta-analysis found that the study design (cross-sectional, longitudinal, controlled comparison, or intervention) does not moderate the relationship between insecure attachment and overall PTSD symptoms [49].

Forty years after the war ended, veterans from the study sample with lifetime PTSD demonstrate insecure attachment patterns. This finding supports the argument that specific psychotherapeutic interventions focusing on attachment organization [52,53,54] should be pursued with these patients, as attachment orientation modification was evident in one PTSD sample after exposure psychotherapy [55]. Such interventions could have consequences both in modifying PTSD symptoms, and also for the formation of a therapeutic relationship, which is also important for recovery. Furthermore, the availability of improving psychotherapeutic interventions for PTSD patients of this age group, such as prolonged exposure therapy, is required—as treatment gains do not appear to be maintained at six-months follow-up [56,57]. Independently of the possible causal relationships between attachment and PTSD, existing studies show that attachment-focused therapeutic interventions can improve PTSD symptoms [58,59]. Additionally, an attachment-directed psychotherapy model has been proposed, which is supported by the relationship between war-related TEs and “moral injury” [53]. Furthermore, one study found that attachment style can predict treatment outcome, and thus improve our knowledge with regards to which psychotherapy works best for whom, and which can be more cost-effective [60]. However, more studies are needed to help us better understand how attachment-focused interventions can be used in clinical practice with PTSD patients.

In our research, we found that total childhood adversity, and particularly, emotional abuse and physical neglect were significantly associated with lifetime PTSD development assessed 40 years after war-related TEs. The association between total childhood adversities and PTSD development was independent of total war experiences.

Another recent cross-sectional study of veterans of the Portuguese colonial wars found that those with PTSD (recruited from the Psychiatry Department of one military hospital) reported significantly higher total ACE scores, and, specifically, a greater level of childhood emotional and physical abuse than those without PTSD (recruited from a snowball sample) [61]. A study of a representative sample of veterans of the same wars found low, but significant correlations between childhood abuse and neglect and PTSD symptoms [32].

Our findings are similar to those of a longitudinal study that assessed ACE before deployment to military conflicts, which found that those who reported ACE in more than one category were at an increased risk of developing post-deployment PTSD—with the strongest association being for physical neglect [26]. Other studies found that the number of childhood traumatic experiences was significantly higher in the group of participants who developed post-deployment PTSD symptoms [62] and that this significantly predicted a high level of PTSD symptoms [23]. ACE could be linked to the increased risk of PTSD development through negative influences on attachment orientations [22], among other causes.

We found that lifetime non-war-related TEs, either before or after war, were not associated with lifetime PTSD, which is contrary to the current belief that prior traumatization [4] and additional life stress [63] are risk factors for developing PTSD. Recent studies have found that prior experience of TEs in the absence of subsequent PTSD development is not a risk factor for PTSD development [64,65]. In our study, prior PTSD was not probable, as all the veterans self-reported good mental health before going to war.

In this study, we specifically assessed TEs as defined by criterion A of the DSM-IV PTSD diagnostic criteria. This criterion enables the evaluation of only those TEs that can cause PTSD, according to DSM-IV. Other studies use a much broader concept of traumatization [66]—or they simply do not define prior TE [67]. In addition, the meta-analysis of Ozer et al. [4] found that the relationship between prior trauma and PTSD was stronger if PTSD resulted from non-combat interpersonal violence, rather than if it resulted from combat exposure.

Combat-related trauma severity is a well-known risk factor for PTSD development [29,63]. We found that all types of war-related experiences were significantly positively associated with lifetime PTSD development, except for the case of war-related experiences of injury or disease. This finding is interesting, as this experience could represent a direct threat to life, although maybe this was experienced by the majority of war veterans, as it meant the end of the war for them. Different results were reported [28], which did not find that combat exposure increases the risk of developing PTSD symptoms, however certain specific war experiences did—such as being wounded or injured and killing an enemy. Further research of this subdomain is warranted, separating injury from disease.

Several recent longitudinal studies have shown similar results to our study. Carrying out a combat role during deployment was significantly associated with probable PTSD [68]. The frequency and intensity of combat were strong predictors of new-onset probable PTSD—specifically the experience of killing [29]. Rona et al. [30] found that combat exposure was a strong and specific predictor of PTSD—especially when involving close contact with the enemy.

In our study, action against civilians was significantly associated with lifetime PTSD development, which is a combat experience that has not been independently reported recently (e.g., [29,30]). This is probably one of the war-related experiences which is most prone to cause moral injuries [31].

We found that attachment patterns did not confound or interact with the association between total war experiences and lifetime PTSD. Another study [9] did not find a moderation role for attachment in the association between intimate partner violence and PTSD, while another [11] did for the anxiety and depend dimensions, but not for the close dimension. These discrepancies could be due to the different methodologies and different TEs assessed. War experiences seem to be strong predictors for lifetime PTSD development in our sample, as they are neither influenced by attachment orientations, nor by childhood adversities.

The biggest strength of our study is the fact that we used a valid “gold standard” instrument to diagnose PTSD. The high cut-off used increases the specificity of the measure.

A longitudinal prospective study would be far more appropriate as it could investigate causality between attachment patterns and PTSD, but is not possible for the population that we studied. On the other hand, assessment more than 40 years after war could be an advantage, due to the delayed onset of PTSD. As we assessed lifetime PTSD 40 years after exposure has occurred, a long time has elapsed during which PTSD can develop, and, albeit possible, it is less probable that new cases will continue to occur. Furthermore, this population is increasing and is in greater need of care.

We did not assess participants’ mental health, in our research, neither working models of attachment before war, and thus could not determine the direction of the associations between PTSD and attachment. Sample size was another limitation. Recall bias might have been a problem in our study, as the colonial wars ended 40 years ago. PTSD symptoms may cause a change in memories of exposure to war [69,70]. However, the way recall bias can influence the associations between war-related TEs and lifetime PTSD is difficult to ascertain in a cross-sectional study, as changes in memories can reflect dissociation or repression of events that did occur, or even the addition of false memories of events that did not occur [71]. Accordingly, childhood adversity memories can also change over such a long time of assessment after their occurrence and can also be changed by war-related trauma and lifetime PTSD. However, a recent review concluded that prospective and retrospective studies of childhood maltreatment identify different groups of mechanisms underlying psychopathology risk and that both have clinical value as risk indicators [72].

We did not study women, and thus the findings cannot be generalized for this population. The same applies to those with non-Caucasian ethnicity. Furthermore, these results cannot also be generalized for non-war-related PTSD.

## 5. Conclusions

Lifetime PTSD for the sample of war veterans studied is significantly associated with insecure attachment, war-related experiences, and childhood adversities. However, this study cannot conclude on the causality of the association between insecure attachment and lifetime PTSD. War-related experiences and specific childhood adversities seem to constitute significant predictors for the development of PTSD, although there is a possibility of recall bias. Severity of war exposure is associated with lifetime PTSD—independently of attachment orientations or childhood adversities. Nevertheless, the significant association of insecure attachment with lifetime PTSD assessed 40 years after war-related TEs supports the importance of attachment-focused interventions for the treatment of war veterans with lifetime PTSD.

## Figures and Tables

**Table 1:** Sociodemographic characteristics of the total sample and according to having or not having lifetime PTSD.

Sociodemographic Characteristic	Total (*n* = 61)	PTSD (*n* = 33)	Non-PTSD (*n* = 28)	*p*
**Age, Years (Mean, SD)**	65.25, 3.37	64.82, 3.41	65.75, 3.30	0.285
Marital Status	*N* (%)	*N* (%)	*N* (%)	
Married	57 (93.4)	32 (97.0)	25 (89.3)	0.325
Divorced or Widow	4 (6.6)	1 (3.0)	3 (10.7)	
**Graffar Index**				
2	8 (13.1)	2 (6.1)	6 (21.4)	0.082
3	38 (62.3)	20 (60.6)	18 (64.3)	
4	15 (24.6)	11 (33.3)	4 (14.3)	
**Disability**				
No	38 (62.3)	23 (69.6)	15 (53.6)	0.195
Yes	23 (37.7)	10 (30.3)	13 (46.4)	
**Territory (Deployment Site)**				
Angola	22 (36.1)	11 (33.3)	11 (39.3)	0.484
Mozambique	19 (31.1)	9 (27.3)	10 (35.7)	
Guinea	20 (32.8)	13 (39.4)	7 (25.0)	

Note: PTSD, posttraumatic stress disorder.

**Table 2:** Association between attachment dimensions and non-war-related traumatic events and lifetime posttraumatic stress disorder—both crude and adjusted.

Attachment and TEs	Unadjusted *OR* (95% CI)	*p*	Adjusted *OR* (95% CI)	*p*
**Attachment**				
Anxiety	3.76 (1.66, 8.52)	0.002	2.34 (0.82, 6.65) ^a^	0.111
Avoidance	18.46 (3.16, 107.72)	0.001	7.21 (1.02, 50.94) ^a^	0.048
Total WEQ score	1.18 (1.07, 1.31)	<0.001	1.17 (1.04, 1.31) ^a^	0.007
Insecure versus Secure	6.37 (1.81, 22.46)	0.002	4.04 (1.00, 16.34) ^b^	0.043
**TEs before War**				
Yes versus no	0.52 (0.13, 2.09)	0.354	-	-
**TEs after War**				
Yes versus no	0.52 (0.19, 1.46)	0.211	-	-

Note: *OR*, odds ratio; CI, confidence interval; WEQ, War Exposure Questionnaire; TEs, traumatic events. ^a^ Model adjusted for attachment anxiety and avoidance and total WEQ score. ^b^ Adjusted for total war exposure.

**Table 3:** Vulnerability factors for lifetime posttraumatic stress disorder development.

Vulnerability Factors	Crude *OR* (95% CI)	*p*	Adjusted *OR* (95% CI)	*p*
**CTQ-SF**				
Total CTQ-SF score	1.06 (1.00, 1.13)	0.037	1.07 (1.00, 1.15) ^a^	0.039
Emotional abuse	1.27 (0.99, 1.63)	0.024	˗	˗
Emotional neglect	1.05 (0.93, 1.18)	0.454	˗	˗
Sexual abuse	0.72 (0.31, 1.69)	0.430	˗	˗
Physical abuse	1.1 (0.87, 1.39)	0.414	˗	˗
Physical neglect	1.23 (1.05, 1.44)	0.004	˗	˗
**War-Related Experiences (WEQ)**				
Total WEQ score	1.18 (1.07, 1.31)	<0.001	1.18 (1.07, 1.31) ^b^	<0.001
War-related experiences	2.05 (1.18, 3.57)	0.004	˗	˗
Physical conditions	1.87 (1.15, 3.06)	0.005	˗	˗
Injury or disease	1.11 (0.80, 1.65)	0.462	˗	˗
Witnessing casualties amongst comrades	1.42 (0.99, 2.04)	0.045	˗	˗
Witnessing casualties amongst the enemy	1.59 (1.17, 2.16)	0.001	˗	˗
Witnessing casualties amongst civilians	1.74 (1.23, 2.47)	<0.001	˗	˗
Action against the enemy	2.03 (1.25, 3.28)	0.002	˗	˗

Note: *OR*, odds ratio; CI, confidence interval; CTQ-SF, Childhood Trauma Questionnaire-Short Form; WEQ, War Exposure Questionnaire. ^a^ Adjusted for total war-related experiences (total WEQ score). ^b^ Adjusted for total childhood adversities (total CTQ-SF score).

## Supplementary Materials

The following are available online at https://www.mdpi.com/2227-9032/8/4/359/s1, Supplementary Materials: War Exposure Questionnaire.
