# Supplementary material for: Vulnerability Factors Associated with Lifetime Posttraumatic Stress Disorder among Veterans 40 Years after War
Source: Healthcare (Basel). 2020 Sep 24;8(4):359. doi: 10.3390/healthcare8040359 (PMC7711782; doi:10.3390/healthcare8040359)
Supplement: Supplementary file 1 [file healthcare-08-00359-s001.pdf]

QUESTIONÁRIO DE EXPOSIÇÃO À GUERRA

Ivone Castro-Vale & Ângela Maia, 2012

Código:__________________

Serviço militar: Obrigatório Voluntário

Início: ____________________ Fim: _________________Ex-colónia: ____________________

Posto militar: Praça Sargento Oficial

Ramo: Exército Força Aérea Marinha **Especialidade:** _________________________

As questões que se seguem referem-se a situações que podem ter acontecido durante a guerra. Por favor indique se lhe aconteceu ou não, e nos casos em que aconteceu, o quanto cada situação o afetou:

Nada	Um pouco	Moderadamente	Bastante	Extremamente
0	1	2	3	4

		não	sim	0	1	2	3	4
**Experiências de guerra:**								
1	Participou em situações de combate em que poderia ter perdido a vida?							
2	Participou em situações de combate em que uma grande parte do seu pelotão perdeu a vida?							
3	Participou em ações de guerrilha?							
4	Foi vítima de ataques com granadas/minas?							
5	Foi vítima de ataques civis?							
6	Foi feito prisioneiro pelo inimigo?							
7	Foi torturado?							
**Condições físicas**								
8	Passou fome/sede?							
9	Teve tanta fome ou sede que comeu/bebeu coisas que nunca pensou comer/beber?							
10	Teve más condições de conforto/higiene ou segurança que o impediam de dormir?							
11	Ficou sem dormir alguns dias seguidos?							
12	O clima era insuportável?							
**Ferimentos e doenças**								
13	Sofreu ferimentos devido a acções de combate?							
14	Sofreu ferimentos por acidente?							
15	Ficou incapacitado? Se sim: Incapacidade: ____%							
16	Sofreu de alguma doença na sequência dos ferimentos?							
17	Foi evacuado para a Metrópole por motivos de doença? Se sim, Qual?_________________________________							

QUESTIONÁRIO DE EXPOSIÇÃO À GUERRA

Ivone Castro-Vale & Ângela Maia, 2012

		não	sim	0	1	2	3	4
	**Assistiu a baixas em companheiros?**							
18	Por ferimento?							
19	Por morte?							
20	Viu um ou vários cadáveres?							
21	Viu companheiros morrer?							
22	Teve de movimentar cadáveres?							
	**Assistiu a baixas no inimigo?**							
23	Por ferimento?							
24	Por morte?							
25	Viu um ou vários cadáveres?							
26	Viu inimigos morrer?							
27	Teve de movimentar cadáveres?							
	**Assistiu a baixas em civis?**							
28	Por ferimento?							
29	Por morte?							
30	Viu um ou vários cadáveres?							
31	Viu civis morrer?							
32	Teve de movimentar cadáveres?							
	**Ação sobre o inimigo:**							
33	Feriu ou matou soldados do inimigo?							
34	Matou 1 ou vários soldados do inimigo para se defender?							
35	Matou 1 ou vários soldados do inimigo sem ser para se defender?							
	**Ação sobre civis:**							
36	Feriu ou matou civis em combate?							
37	Participou em chacinas de civis?							
38	Foi obrigado a ferir ou matar civis para obedecer a ordens?							
